# Prognostic value of S100A4 and Glypican-3 in hepatocellular carcinoma in cirrhotic HCV patients

**DOI:** 10.1186/s43046-023-00184-1

**Published:** 2023-08-21

**Authors:** Mahmoud El-Bendary, Khaled Farid, Mohammad Arafa, Wagdi Elkashef, Talaat Abdullah, Ahmed El-Mesery

**Affiliations:** 1 Tropical Medicine and Hepatogastroenterology Department, Mansoura Faculty of Medicine, Mansoura University, Mansoura, 35516 Dakahlyia Egypt; 2 Pathology Department, Mansoura Faculty of Medicine, Mansoura University, Mansoura, Egypt; 3 Gastroenterology Surgery Center, Mansoura Faculty of Medicine, Mansoura University, Mansoura, Egypt

**Keywords:** Hepatocellular carcinoma, Cirrhotic, HCV, Prognosis, S100A4, Glypican-3

## Abstract

**Aims:**

Both S100A4 and Glypican-3 have been known to be engaged in HCC development and progression. This study aimed to evaluate both S100A4 and GPC3 expression in HCC tissues as a prognostic markers.

**Methods:**

Tissues from 70 patients of HCC in cirrhotic HCV patients were evaluated by immunohistochemistry using antibodies against SA100A4 and GPC3 and compared with tumor-adjacent tissue (controls). All cases were followed for 40 months.

**Results:**

GPC3 was more expressed in HCC (79%) than S100A4 (21%). S100A4 was more significantly expressed in cases showing metastasis, microscopic vascular emboli, necrosis, and grade III tumors. There was no relationship between overall survival and both S100A4 and GPC3. The only significant independent predictor for recurrence was decompensation (*OR* 3.037), while metastasis was significantly predicted by S100A4 expression (*OR* 9.63) and necrosis (*OR* 8.33).

**Conclusion:**

S100A4 might be used as a prognostic marker for HCC, while GPC3 is a reliable marker of HCC diagnosis.

## Introduction

Hepatocellular carcinoma (HCC) is one of the most unfavorable malignancies worldwide. HCC ranked globally from third to second cancer. It is currently the second leading cause of cancer-related deaths in men and the fifth in women [1]. The overall 5-year survival rate for HCC is only 5%. The relapse rate within 5 years of undergoing surgery is 70%, and the recurrence rate within the remaining liver tissue is more than 80%. Although there are similar clinicopathological parameters in HCC patients, the outcome is entirely different, confirming the unsolved biological behavior of this tumor [2]. In Egypt, HCV is considered to be a significant risk factor for HCC [3, 4].

Expression of S100A4 is related to epithelial mesenchymal transition (EMT). EMT is a process of tumor-cell invasion leading to loss of epithelial characteristics and acquiring mesenchymal features. In HCC, S100A4 expression, together with other EMT-related proteins, is equivalent to metastasis and overall survival [5]. A more vital metastatic ability has been reported in S100A4-expressing tumor cells than other S100A4-negative cells [6]. The expression of S100P gradually progressed from dysplasia, precancerous lesions to HCC. S100P may be a contributing factor for HCC formation or progression. Although S100P expression is well-known to be linked to many neoplastic disorders, its role in HCC has not been extensively studied. Malignant hepatocyte cell lines express higher S100P levels than normal nonmalignant cells, which lack S100P [7]. Glypican-3 (GPC3) is one of the members of the heparin sulfate (HS) proteoglycan family. GPC3 is strikingly expressed in human embryos regulating morphogenesis probably depending on insulin-like growth factor, bone morphogenic protein, fibroblast growth factor (FGF), or hedgehog signaling. GPC3 is profusely expressed during pregnancy in fetal organs such as the liver, lung, kidney, and placenta. On the other hand, GPC3 expression is reduced in adults [8]. GPC3 is highly expressed in early HCC, so it is a sensitive and specific biomarker for diagnosis. Moreover, GPC3expression was proved as a prognostic factor and could predict the poor outcome for HCCs [9].

The aim of this study is to evaluate the prognostic value of tissue expression of both S100A4 protein and GPC3 in HCC in Egyptian patients with HCV cirrhosis.

## Materials and methods

### Patients

Only 70 out of 400 patients with primary HCC (56 males and 14 females) with a mean age 63.03 ± 8.17 years were examined and investigated at both Tropical Medicine Department and Gastroenterology Surgery Center Mansoura University between January 2016 and November 2019. The 70 patients met the inclusion criteria for hepatectomy and were recruited in the current study. Laboratory investigations were conducted in the Molecular Genetic Unit of Endemic Hepatogastroenterology and Infectious Diseases (MGUHID) of Mansoura Faculty of Medicine. This study was approved by the Institutional Review Board of Mansoura Faculty of Medicine, and written informed consent was obtained from all patients. Diagnosis of HCC depended on one dynamic imaging technique for lesions beyond 2 cm in diameter with the typical hypervascular pattern in the arterial phase with washout in the portal venous or delayed phases. For the diagnosis of lesions less than 2 cm, two dynamic techniques were obtained [10]. Patients who met all of the following criteria were included in the analysis: (i) patients with Child-Pugh class A, (ii) no main portal vein branch thrombosis or extrahepatic metastasis, (iii) prothrombin concentration greater than 50%, (iv) platelet count greater than 50.000/mm^3^, and (v) accepted performance status for hepatectomy.

**Fig. 1:**
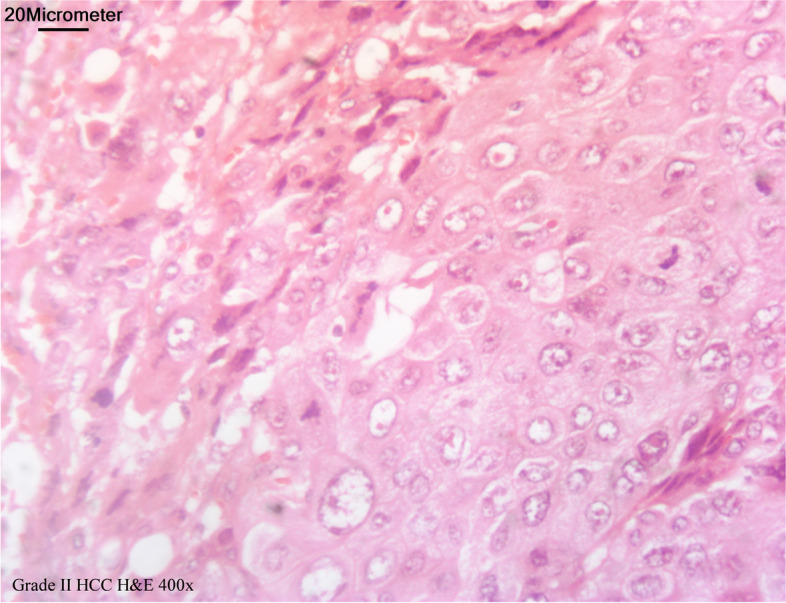
HCC grade II hematoxylin and eosin (400×)

Clinical data were collected, including age, gender, complete blood count, prothrombin time, albumin, bilirubin, *α*-fetoprotein (AFP), and alkaline phosphatase. Tissue samples were taken from resection specimens from both tumorous tissue and adjacent para-tumorous cirrhotic tissue (control). Diagnosis of HCC in all cases was confirmed histopathologically (Fig. 1). Moreover, pathological data were collected, including tumor size, location, number, grading, growth pattern, mitotic count, necrosis, and microscopic vascular emboli. Follow-up of the patients was done for 40 months to detect local recurrence, metastasis, and/or decompensation.

### Tissue microarray

**Fig. 2:**
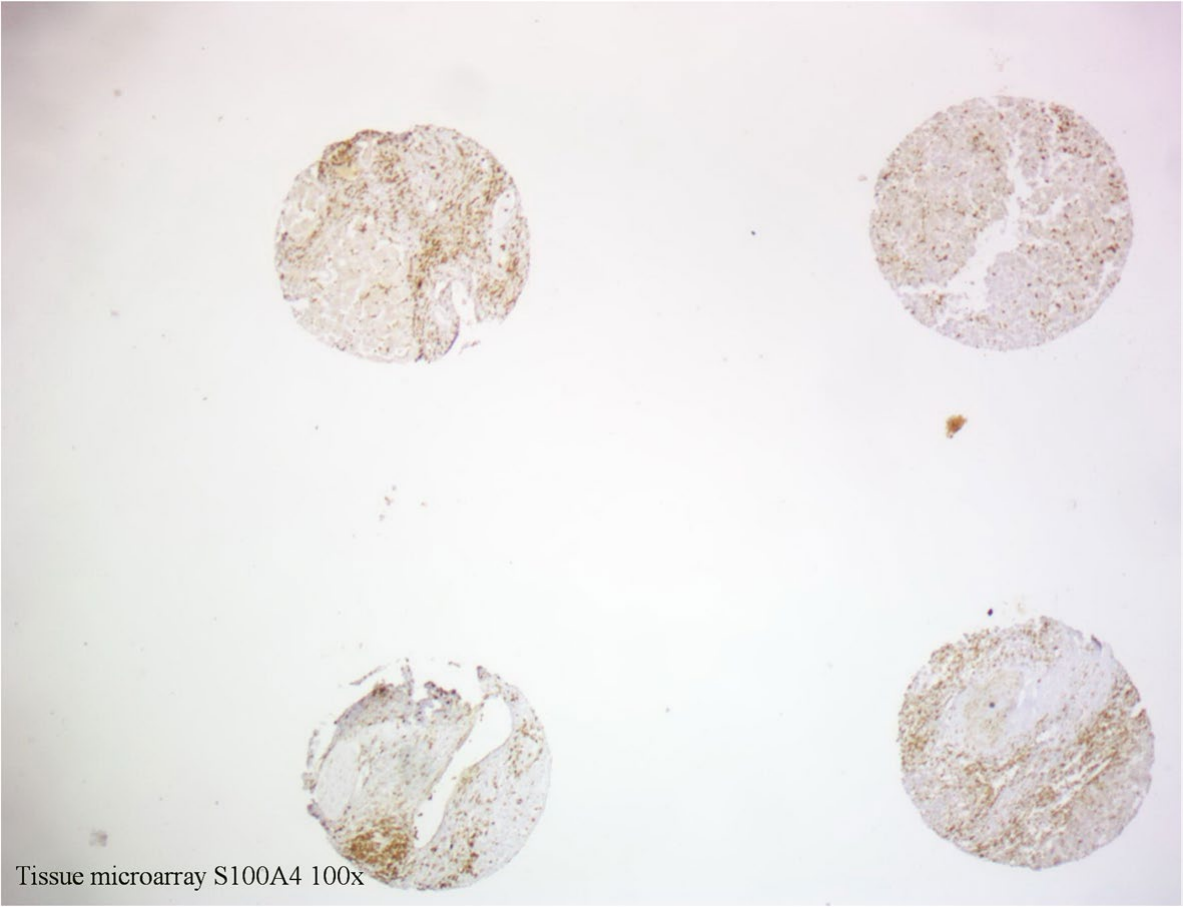
TMA S100A4 (100×)

After selecting areas for construction of TMAs on the donor blocks, sampling was done using a manual arraying instrument (Beecher Instruments, Sun Prairie, WI, USA). Four TMA blocks were constructed using 1-mm tissue cores (Alphelys, Plaisir, France). The blocks were cut into 5-μm sections and coated with paraffin for future use (Fig. 2).

### Immunohistochemistry

Mouse monoclonal antibodies against SA100A4 (Novus Biologicals USA) and GPC3 (1G12; Cell Marque, Rocklin, CA, USA) were used for IHC on the slides of TMAs. The staining procedures were performed according to the manufacturers’ instructions. Two independent investigators examined all samples. The slides were examined using Olympus CX41 microscopy, and photos were taken using Amscope MU1000 camera. Positive staining for S100A4 protein and GPC3 was observed as yellow or brown staining in the cytoplasm of HCC tumor cells. Samples were categorized into one of two groups, based on the level of immune-staining: positive:$\ge$ 5%of cells stained and negative: < 5% of cells stained.

### Statistical analysis

Data were analyzed with *SPSS* version 21. The normality of data was first tested with a one-sample Kolmogorov-Smirnov test. Qualitative data were described using numbers and percentages. Association between categorical variables was tested using the chi-square test and Fisher exact test. Continuous variables were presented as mean ± SD (standard deviation) for parametric data and median for nonparametric data. The two groups were compared with the Student *t*-test (parametric data) and Mann-Whitney test (nonparametric data). *p*-value is considered significant when ≤ 0.05. The markers with *p* < 0.05 were analyzed by stepwise logistic regressions to evaluate independent variables. Survival was expressed in months, and survival analysis was performed by the Kaplan-Meier method with log-rank analysis.

## Results

### Clinicopathological characteristics of the study group

The clinicopathological features of patients in the study group are summarized in Table 1. Microscopic vascular emboli were present in 26 patients (37.1%). Necrosis within tumor tissue was present in 29 cases (41.4%). The main growth pattern was trabecular and was found in 37 cases (52.9%). Local recurrence was reported in 17 cases (24.3%), metastasis in 6 cases (8.6%), and decompensation in 20 cases (28.6%). The median survival time was 7 months (range: 1–40 months).

**Table 1:** Pathological characteristics of the study group

**Variables**	**Study group (*n* = 70)**	
	No	%
**Lobe**		
RT	27	38.6
LT	43	61.4
**Size**	6.40 ± 2.82	
**Pathological parameters**		
**Necrosis**		
Yes	29	41.4
No	41	58.6
**Microscopic vascular emboli**		
Yes	26	37.1
No	44	62.9
**Grading**		
1	37	52.9
2	19	27.1
3	14	20.0
**Mitotic count**	Median = 2, min-max (1–54)	
**Decompensation**		
Yes	20	28.6
No	50	71.4
**Overall survival**		
Median (min-max)	7 (1–40)	
**Local recurrence**		
Yes	17	24.3
No	53	75.7
**The onset of local recurrence after resection**		
Median (min-max)	3 (1–36)	
**Metastasis**		
Yes	6	8.6
No	64	91.4
**Growth pattern**		
Trabecular	40	57.1
Trabecular & acinar	25	35.7
Diffuse	2	2.9
Pseudo acinar	2	2.9
Solid	1	1.4

### Immunohistochemistry of S100A4 in liver tissues

**Fig. 3:**
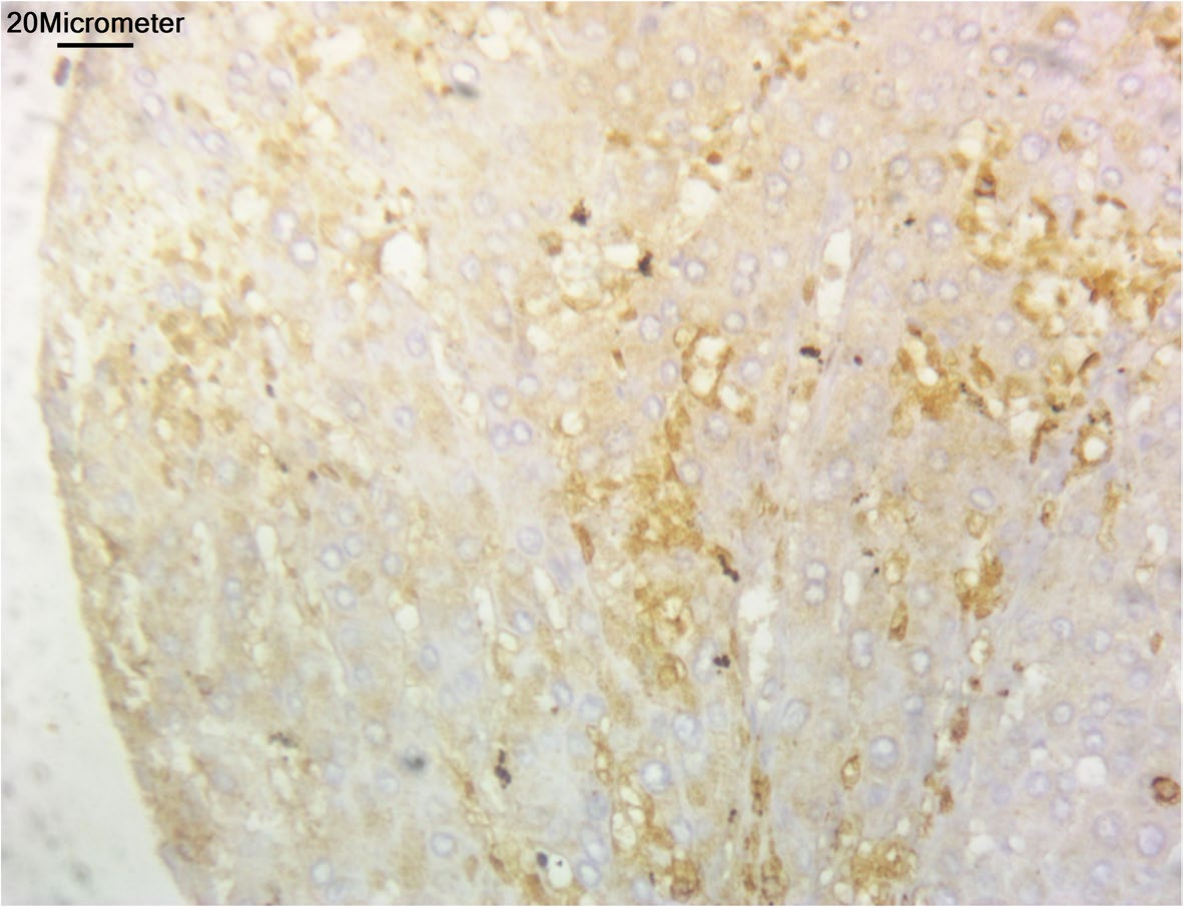
Positive S100A4 showing mild diffuse cytoplasmic reaction (brown)

**Fig. 4:**
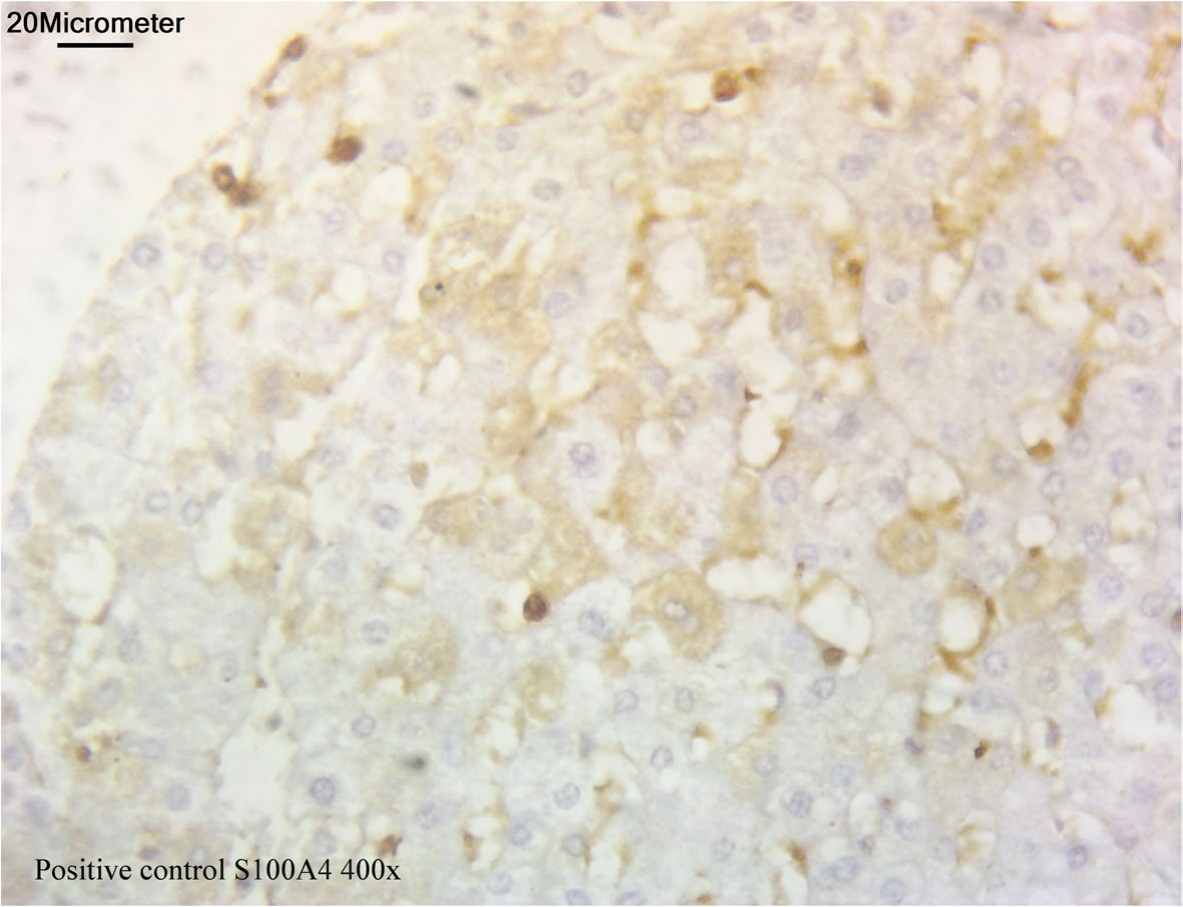
Positive S100A4 showing mild diffuse cytoplasmic reaction (brown) (400×)

HCC tissues showed more staining intensity for S100A4 protein than their tumor-adjacent tissue (control) (Figs. 3 & 4) as 21% (15/70) of HCC cases were positive for S100A4 protein, whereas only one weak positive control was observed (4.2%, *p* = 0.001).

### Significance of S100A4 protein detection in HCC tissues

The detection of S100A4 protein in HCC tissue was found to be significantly associated with metastasis (26.7% versus 3.6% (*p* < 0.005), microscopic vascular emboli (60% versus 30.9% (*p* = 0.039)), necrosis (66.7% versus 34.5% (*p* = 0.025), high-grade tumors (*p* = 0.014), and higher AFP levels (*p* = 0.004). No significant association was observed between S100A4 expression and HCC tumor size, local recurrence, rate of decompensation, or survival time (*p* > 0.05) (Table 2).

**Table 2:** Relation between both S100A4 and GPC3 and other variables

**Variables**	**S100A4**						**Glypican 3**					
	**Positive (*n* = 15)**		**Negative (*n* = 55)**		***χ***^**2**^	***p*-value**	**Positive (*n* = 55)**		**Negative (*n* = 15)**		***χ***^**2**^	***p*-value**
	No.	%	No.	%			No.	%	No.	%		
**Age**												
≤ 60 y	6	40.0	19	34.5	0.610	0.737	19	34.5	6	40.0	2.468	0.291
> 60–70 y	6	40.0	28	50.9			29	52.7	5	33.3		
> 70 y	3	20.0	8	14.5			7	12.7	4	26.7		
Mean ± SD	61.46 ± 9.71		63.45±7.74		*t* = 0.833	0.408	62.92 ± 6.92		63.40 ± 11.99		t= 0.197	0.844
**Sex**												
Male	14	93.3	42	76.4	2.121	0.145	42	76.4	14	93.3	2.121	0 .145
Female	1	6.7	13	23.6			13	23.6	1	6.7		
**Decompensation**												
Yes	4	26.7	16	29.1	0.034	0.854	18	32.7	2	13.3	2.172	0.141
No	11	73.3	39	70.9			37	67.3	13	86.7		
**Overall survival**	9 (1–40)		6 (1–40)		*Z* = 0.804	0.421	5.5 (1–40)		8.5 (2–40)		*Z* = 1.222	0.222
**Local recurrence**												
Yes	2	13.3	15	27.3	1.245	0.264	14	25.5	3	20.0	0.191	0.662
No	13	86.7	40	72.7			41	74.5	12	80.0		
**The onset of local recurrence after resection**	20.5 (5–36)		3 (1–32)		*Z* = 1.372	0.235	2.5 (1–18)		32 (9–36)		*Z* = 2.32	0.02*
**Metastasis**												
Yes	4	26.7	2	3.6	7.977	0.005*	3	5.5	3	20.0	FET	0.074
No	11	73.3	53	96.4			52	94.5	12	80.0		
**Necrosis**												
Yes	10	66.7	19	34.5	5.011	0.025*	21	38.2	8	53.3	1.115	0.291
No	5	33.3	36	65.5			34	61.8	7	46.7		
**Microscopic vascular emboli**												
Yes	9	60.0	17	30.9	4.272	0.039*	19	34.5	7	46.7	0.742	0.389
No	6	40.0	38	69.1			36	65.5	8	53.3		
**Grading**												
1	5	33.3	32	58.2	8.523	0.014*	29	52.7	8	53.3	0.784	0.676
2	3	20.0	16	29.1			16	29.1	3	20.0		
3	7	46.7	7	12.7			10	18.2	4	26.7		
**Size**	7.38 ± 4.28		6.13 ± 2.25		*t* = 1.485	0.143	6.21 ± 2.68		7.15 ± 3.33		*t* = 1.062	0.292
**AFP**	625 (15–2000)		27 (2.2–2000)		*Z* = 2.852	0.004*	45 (2.2–2000)		10 (2.2–1283)		*Z* = 2.056	0.04*
**AST**	65 (24–104)		72 (20–190)		*Z* = 0.791	0.429	71.50 (20–190)		65 (20–119)		*Z* = 0.677	0.499
**ALT**	42.5 (21–94)		55 (20–200)		*Z* = 1.246	0.213	49.5 (21–200)		54 (20–130)		*Z* = 0.662	0.508

*χ*^2^ chi-square test, *t* Student *t*-test, *Z* Mann-Whitney test, *FET* Fisher exact test

*p value >0.05% and is statistically significant

### Immunohistochemistry of GPC3 in liver tissues

**Fig. 5:**
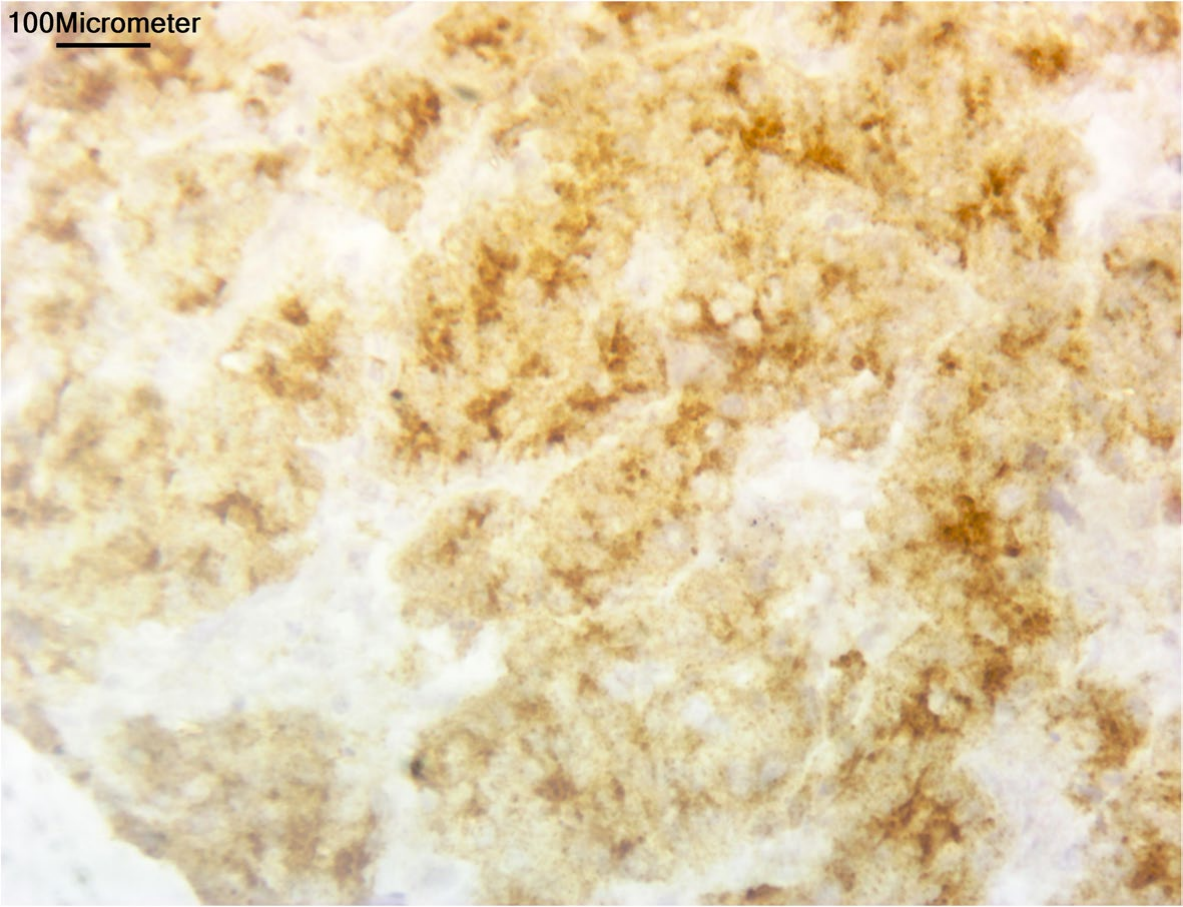
Positive GPC3 showing diffuse cytoplasmic granular reaction (brown) (400×)

GPC3 was more expressed in HCC samples than in tumor-adjacent tissue (control) samples (Fig. 5) as 79% (55/70) of HCC tissues showed positive staining for GPC3, while only 4.2% (3/70) of tumor-adjacent tissues (control) had weak and focal staining of duct epithelium (but not hepatocytes) (*p* < 0.001).

### Significance of GPC3 expression in HCC tissues

Detection of GPC3 in HCC tissues was found to be associated with short tumor-free survival and rapid recurrence (*p* = 0.02). Moreover, the levels of AFP were higher in positive cases than in negative cases (*p* = 0.04). No significant association was present between GPC3 expression and patient survival, tumor metastasis, or recurrence rate (*p* > 0.05). Also, no association was observed with tumor grading, necrosis, microscopic vascular emboli, or tumor size (*p* > 0.05) (Table 2).

### Logistic regression analysis

Stepwise logistic regression analysis was applied to evaluate variables independently to predict tumor recurrence. Only decompensation was found to predict recurrence (*p* = 0.05) (*OR* 3.037) (Table 3).

**Table 3:** Logistic regression analysis of independent predictors for recurrence

Independent predictor	*β*	*P*	OR (95% *CI*)
**Decompensation** No (*r*) Yes	1.111	0.058	3.037 (0.962–9.58)
Constant Model*χ*^2^ % correctly predicted	−1.516 3.45, *p* = 0.06 75.7%		

Many variables were found to be associated with tumor metastasis: S100A4, necrosis, size of the tumor, AST level, and ALT level (*p* = 0.017, 0.029, 0.03, 0.03, and 0.006, respectively). After univariate regression analysis, S100A4 expression (*OR* 9.63) and necrosis (*OR* 8.33) were independent predictors. After multivariate regression analysis, S100a4 expression was found to be the most significant predictor of tumor metastasis (*OR* 8.4) (Table 4).

**Table 4:** Logistic regression analysis of independent predictors for metastasis

Independent predictor	Univariate regression			Multivariate regression	
	*β*	*P*	OR (95% *CI*)	*P*	AOR (95% *CI*)
**S100A4**	2.266	0.015*	9.63 (1.56–59.3)	0.041*	8.4 (1.08–49.8)
Negative (*r*)					
Positive					
**Necrosis**	2.120	0.048*	8.33 (1.08–75.6)	-	-
No (*r*)					
Yes					
**Size**	−0.562	0.078	0.570 (0.3–1.06)	-	-
**AST**	−0.047	0.056	0.95 (0.9–1.001)	-	-
**ALT**	−0.097	0.05*	0.91 (0.82–1)	0.066	0.9 (0.8–1.01)
Constant	0.771				
Model*χ*^2^	12.17, *p* = 0.002				
% correctly predicted	95.3%				

*COR* crude *OR*, *AOR* adjusted OR

*p value >0.05% and is statistically significant

### Kaplan-Meier log-rank analysis of survival

**Fig. 6:**
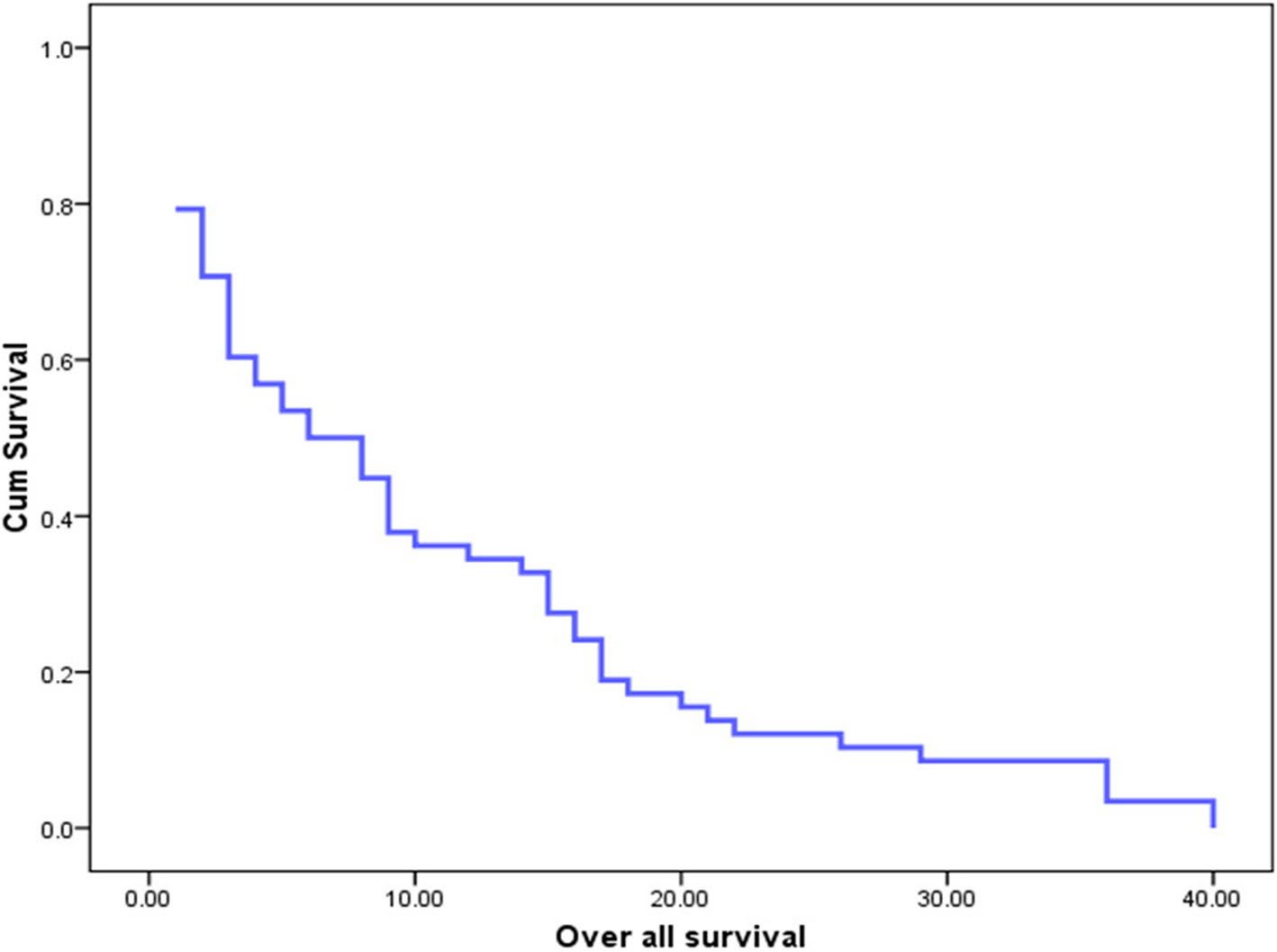
Kaplan-Meier curve for survival

The cumulative percentage for survival at 6, 12, 18, 24, 30, 36, and 40 months was 55.7, 15.9, 14.3, 4.3, 2.9, 4.3, and 2.9, respectively (Fig. 6). The median overall survival was shorter in decompensated patients and higher-grade tumors (*p* = 0.012 and *p* = 0.046, respectively) (Table 5).

**Table 5:** Relation between overall survival and clinicopathological variables

	**Median overall survival (95% *CI*)**	**Log rank (Mantel-Cox)**	***p*-value**
**Sex**			
Male	6 (2–9.9)	0.276	0.600
Female	3 (0–21)		
**S100**			
Positive	9 (1.7–16.2)	0.585	0.444
Negative	6 (1.9–10.05)		
**Glypican**			
Positive	5 (0–10.6)	1.327	0.249
Negative	8 (3.3–12.6)		
**Decompensation**			
Yes	2 (0.2–3.7)	3.79	0.012*
No	9 (7.5–10.5)		
**Recurrence**			
Yes	6 (0–15.4)	0.381	0.537
No	8 (3.8–12.15)		
**Metastasis**			
Yes	3 (0–6.6)	0.165	0.684
No	8 (4.4–11.5)		
**Necrosis**			
Yes	4 (1.5–6.4)	1.835	0.176
No	9 (5.3–12.7)		
**Microscopic vascular emboli**			
Yes	9 (1.6–16.4)	0.037	0.848
No	6 (2.02–9.9)		
**Grading**			
1	10 (2.7–17.3)	6.164	0.046*
2	3 (0.6–5.35)		
3	5 (0–12.5)		

*p value >0.05% and is statistically significant

## Discussion

Hepatocellular carcinoma (HCC) is the most common form of liver malignancies worldwide [4, 11]. There are many risk factors for HCC recurrence and progression, including tumor size, associated cirrhosis, HBV infection, HCV infection, histological grade of tumor differentiation, vascular permeation, and the absence of capsule [12–14].

The molecular pathogenesis of HCC disease remains mysterious even after both causative factors and cellular changes have been recognized [15–18]. Furthermore, therapeutic modalities for HCC remain limited. More interpretation of the underlying biology of HCC is needed for further development in subsequent targeted therapies against essential molecular mechanisms of HCC [19].

S100A4 and other S100P (proteins) have been studied in many in vitro studies on HCC cell lines, revealing higher S100P levels than normal nonmalignant hepatocytes, which frequently lack S100P. Also, silencing of endogenous S100P was observed to decrease HCC cell growth by Kim et al. [7].

The present study reported that expression of S100A4 in HCC patients is associated with metastasis, higher grades of HCC, microscopic vascular embolization, and tumor necrosis. These findings may implicate that more biologically aggressive tumors are related to the positivity of S100A4 in tumor tissues.

Cui et al. (2006) reported that S100A4 was overexpressed only in the metastatic cells among other metastasis candidate proteins in HCC cell lines and was selected for further series of assays. The authors suggested that S100A4 might contribute to HCC invasion and metastasis through two matrix metalloproteinase (MMP9) secretion regulation paths and strengthened motility and invasion properties [6].

Yan et al. (2013) reported that S100A4 expression was significantly higher in liver cancer-associated mesenchymal stem cells (LC-MSCs) compared with liver normal MSCs (LN-MSCs) from adjacent cancer-free tissues. They also revealed that S100A4 secreted from LC-MSCs has a role in HCC progression and may be a potential therapeutic target [20].

In a study on mainly HBV patients, Liu et al. found that S100A4 correlated with tumor differentiation, invasion, recurrence, and overall survival and concluded that it could be a valuable marker of tumor aggressiveness and prognosis [21].

In the current study, there was a significant positive correlation between AFP level and S100A4 expression. At the same time, Liu et al. found no significant association between S100A4 expression and AFP level [21].

On the other hand, Cho et al. found that S100A4 expression in HCC was not statistically correlated with clinicopathologic parameters, including histologic grade, stage, capsular invasion, intrahepatic metastasis, and portal vein invasion. Also, they found that gene amplification of S100A4 was not associated with clinical parameters [22].

Sample sizes, population admixture, differences in ethnic backgrounds, differences in etiology, and different criteria for selection of target population may all contribute to these conflicting results and discrepancies.

The primary etiology of liver disease in HCC patients significantly impacts patient outcome and cannot be neglected as a cofactor with other variables [23, 24]. Many studies concluded that survival is significantly better in HCV-related HCC than in HBV-related HCC. Cantarini et al. reported that hepatitis C virus-related HCC has a lesser aggressiveness than hepatitis B virus-related HCC, as HCV-related HCC becomes clinically manifest once they have reached an advanced stage [17, 25]. Sinn et al. concluded that HCV-related HCC has a better prognosis than HBV [26]. The discrepancy in survival has also been previously described in breast cancer studies with S100A4. The discrepancy has been attributed to fewer case numbers, the difference in stages of disease included in different studies, a relatively short period of follow-up, and differences in sample storage conditions or fixation methods [27, 28].

GPC3 has been established as a diagnostic marker for HCC. Further studies have evaluated its value in prognosis suggesting its role as a promising prognostic biomarker. Many meta-analytic studies confirmed that GPC3 expression is of prognostic value as it was correlated with shorter overall survival (OS) and disease-free survival (DFS) of HCC patients [29].

In the current study, the diagnostic value of GPC3 in HCC has been confirmed, supporting its established value. However, regarding its prognostic value, its expression was only correlated with the time of recurrence of HCC, and there was no correlation with patient overall survival. Similar results were obtained with Chen et al., who revealed that GPC3 expression was a significant independent prognostic factor for disease-free survival. However, overall survival was not affected, but this study included only cases of early HCC [29].

Unlike most previous studies, the present study found no association between GPC3 and tumor differentiation and other clinicopathological parameters [29].

This conflict between this study and previous studies might be explained by some limitations which were addressed in some of those studies. First, positive results were more published than negative ones, suggesting a potential risk of bias. In addition, the total number of included studies and the total sample size were relatively small, which might influence the validity of the analysis to some extent [29]. Moreover, many of these studies were based on the Asian population. Also, there was a lack of data about other races. In addition, the IHC analysis technique has many related factors such as the type of antibody used, detection method, evaluation method of results, and interobserver variation that lead to the heterogeneity of IHC studies [29].

There are a few limitations to this study. All the patients were HCV-induced HCC, and no comparison was made with other causes of liver disease. The small sample size (70 patients), a short period of follow-up, and operative complications may have an impact on survival, and it was not clarified here. Moreover, in patients who had decompensated liver disease, there was a defect in follow-up for recurrence of HCC, suggesting a possibility of missed recurrence or metastasis in this group of patients.

## Conclusion

This study suggests that S100A4 protein levels may be a valuable tool in predicting poor outcomes in HCC, and this may play a role as a therapeutic target in the future. Additionally, it is recommended that patients with positive S100A4 expression may need more close follow-up and more intensive treatment modalities. We also confirmed the diagnostic value of GPC3 in HCC.

### Limitation of the study

Small number size of the HCC patients and the absence of the Western blot technique are considered some of the limiting factors in this study.

## Data Availability

The datasets used and/or analyzed during the current study are available from the corresponding author on reasonable request.

## Abbreviations

HCC: Hepatocellular carcinoma
GPC3: Glypican-3
EMT: Epithelial mesenchymal transition
S100A4: S100 calcium-binding protein A4
HS: Heparin sulfate
FGF: Fibroblast growth factor
MGUHID: Molecular genetic unit of endemic hepatogastroenterology and infectious diseases
AFP: α-Fetoprotein
MMP9: Matrix metalloproteinase
LC-MSCs: Liver cancer-associated mesenchymal stem cells
IHC: Immunohistochemistry
